# An Aeromagnetic Compensation Method Based on a Multimodel for Mitigating Multicollinearity

**DOI:** 10.3390/s19132931

**Published:** 2019-07-03

**Authors:** Guanyi Zhao, Qi Han, Xiang Peng, Pengyi Zou, Haidong Wang, Changping Du, He Wang, Xiaojun Tong, Qiong Li, Hong Guo

**Affiliations:** 1 School of Computer Science and Technology, Harbin Institute of Technology, Harbin 150001, China; 2 State Key Laboratory of Advanced Optical Communication Systems and Networks, Department of Electronics, and Center for Quantum Information Technology, Peking University, Beijing 100871, China; 3 Hangzhou Applied Acoustic Institute, Hangzhou 310023, China

**Keywords:** magnetometer, aeromagnetic survey, aeromagnetic compensation, linear regression, multicollinearity

## Abstract

Aeromagnetic surveys play an important role in geophysical exploration and many other fields. In many applications, magnetometers are installed aboard an aircraft to survey large areas. Due to its composition, an aircraft has its own magnetic field, which degrades the reliability of the measurements, and thus a technique (named aeromagnetic compensation) that reduces the magnetic interference field effect is required. Commonly, based on the Tolles–Lawson model, this issue is solved as a linear regression problem. However, multicollinearity, which refers to the case when more than two model variables are highly linearly related, creates accuracy problems when estimating the model coefficients. The analysis in this study indicates that the variables that cause multicollinearity are related to the flight heading. To take this point into account, a multimodel compensation method is proposed. By selecting the variables that contribute less to the multicollinearity, different sub-models are built to describe the magnetic interference of the aircraft when flying in different orientations. This method restricts the impact of multicollinearity and improves the reliability of the measurements. Compared with the existing methods, the proposed method reduces the interference field more effectively, which is verified by a set of airborne tests.

## 1. Introduction

Aeromagnetic surveys originated in the 1930s for military applications [1] and now play a very important role in many other fields, such as geophysical exploration. With the magnetometers installed aboard aircrafts, people can measure the magnetic field at a very flexible scale [2]. However, since the aircraft has its own magnetic field, which degrades the reliability of the measurements, a technique to reduce the magnetic interference field is required, that is, aeromagnetic compensation [3,4].

Aeromagnetic compensation is mainly based on the Tolles–Lawson (T–L) model [5,6], which decomposes the magnetic interference field into three sources: the permanent field, induced field, and eddy-current field. Combining all of these fields, the magnetic interference field can be described as a linear equation with 18 or 16 terms (the latter being simplified from an 18-term equation) [7]. Solving this equation is called a calibration, which is a key issue in aeromagnetic compensation, and the elements of the solution are called coefficients. Commonly, a figure-of-merit (FOM) flight [8] is implemented for calibration, which includes four orthogonal headings and three sets of maneuvers (pitches, rolls, and yaws) in each heading. After removing the ambient magnetic field from the measured total field, the remaining field is considered to be the magnetic interference field, which can be the dependent variable of the 16-term (or 18-term) linear equation. Then the coefficients can be estimated through regression.

To yield a more accurate set of coefficients, researchers have made many efforts toward improving the model [9,10], optimizing the solving method [11,12,13,14,15,16], and correcting the sensor errors [17]. Theoretically, with the assumption that the ambient magnetic field is uniform [7], the coefficients of the T–L model are directly linked to the aircraft itself because they are due to its properties, such as the materials and the electrical systems. However, in practice, the coefficients are hard to obtain accurately. One reason for this is that the uniformity assumption of the ambient magnetic field is unrealistic. Another significant factor is multicollinearity among the 16 or 18 variables of the linear equation, which causes noise sensitivity in the estimation. To mitigate the multicollinearity, some statistical methods are utilized, such as ridge regression (RR) [7], truncated singular value decomposition (TSVD) [14], and partial least-squares regression (PLSR) [15]. These methods render the estimated coefficients more accurate for the 18-term model but are not always effective for the 16-term model [7]. Two variables are excluded in the 16-term model because they can be linearly represented by other variables and contribute significantly to the multicollinearity. Nevertheless, the multicollinearity is typically still strong.

In this paper addressing aeromagnetic compensation based on scalar magnetometers, we analyze the sources of multicollinearity and find those that depend on the flight heading. Differing from the present methods that regard the FOM flight as a whole, here we propose a multimodel method to compensate for the magnetic interference field of the aircraft, according to the flight heading. By selecting different variable sets for different headings, multicollinearity can be inhibited.

This paper is structured as follows. In Section 2, we describe the T–L model, analyze why multicollinearity occurs, and present our method. In Section 3, the method’s performance is verified by a set of airborne tests. Section 4 is the conclusion.

## 2. Analysis and Method

### 2.1. T–L Model

**Figure 1:**
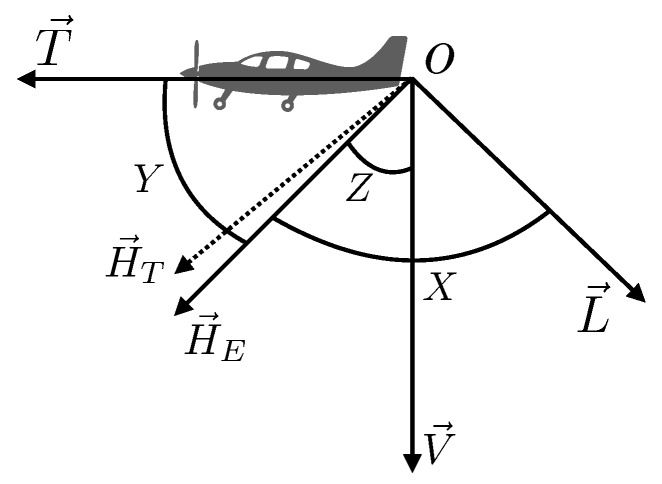
The LTV reference system.

To better describe the analysis and the method, the T–L model is briefly introduced here. First, the reference system is displayed in Figure 1. The reference system is attached to the aircraft: the origin *O* is at the location of the magnetometer, the *L*-axis is parallel to the left, the *T*-axis points toward the nose, and the *V*-axis is vertical. The vector ${\vec{H}}_{E}$ represents the magnetic field of the earth, and *X*, *Y*, and *Z* are the angles between the magnetic field of the earth and the three axes, respectively. The vector ${\vec{H}}_{T}$ represents the total field measured by the magnetometer. As scalar magnetometers only measure the intensity of the total magnetic field and as the intensity of the earth magnetic field is a few orders greater than that of the interference field, we can define the scalar of the interference field as the projection from itself onto the direction of the earth’s magnetic field.

According to the T–L model [6], the magnetic interference field can be decomposed into three parts: the permanent field, the induced field, and the eddy-currents field. The permanent field is constant, written as $\left(\begin{array}{ccc} a_{1} & a_{2} & a_{3} \end{array}\right)$, the scalar of which can be represented as

(1) $$H_{p}=\frac{\vec{H_{p}}\cdot \vec{H_{E}}}{H_{E}}=\left(\begin{array}{ccc} a_{1} & a_{2} & a_{3} \end{array}\right)\left(\begin{array}{c} \cos X\\ \cos Y\\ \cos Z \end{array}\right).$$

The second part of the magnetic interference field is induced by the magnetic field of the earth and can be represented as

(2) $$H_{i}=\frac{\vec{H_{i}}\cdot \vec{H_{E}}}{H_{E}}=H_{E}\left(\begin{array}{ccc} \cos X & \cos Y & \cos Z \end{array}\right)\left(\begin{array}{ccc} b_{11} & b_{12} & b_{13}\\ b_{21} & b_{22} & b_{23}\\ b_{31} & b_{32} & b_{33} \end{array}\right)\left(\begin{array}{c} \cos X\\ \cos Y\\ \cos Z \end{array}\right).$$

The third part is caused by the eddy currents in the aircraft and can be expressed as

(3) $$H_{e}=\frac{\vec{H_{e}}\cdot \vec{H_{E}}}{H_{E}}=H_{E}\left(\begin{array}{ccc} \cos\dot{X} & \cos\dot{Y} & \cos\dot{Z} \end{array}\right)\left(\begin{array}{ccc} c_{11} & c_{12} & c_{13}\\ c_{21} & c_{22} & c_{23}\\ c_{31} & c_{32} & c_{33} \end{array}\right)\left(\begin{array}{c} \cos X\\ \cos Y\\ \cos Z \end{array}\right),$$

where $\cos\dot{X}$, $\cos\dot{Y}$, and $\cos\dot{Z}$ are the time derivatives of cosX, cosY, and cosZ, respectively, which are defined as

(4) $$\cos\dot{W}=\frac{d\cos W(t)}{dt},$$

where *W* represents *X*, *Y*, or *Z*, which varies with time. Thus, the magnetic interference is expressed as

(5) $$\begin{array}{ccc} H_{I} & = & H_{p}+H_{i}+H_{e}\\ & = & a_{1}\cos X+a_{2}\cos Y+a_{3}\cos Z\\ & & +H_{E}b_{11}\cos^{2}X+H_{E}\left(b_{12}+b_{21}\right)\cos X\cos Y+H_{E}\left(b_{13}+b_{31}\right)\cos X\cos Z\\ & & +H_{E}b_{22}\cos^{2}Y+H_{E}\left(b_{23}+b_{32}\right)\cos Y\cos Z+H_{E}b_{33}\cos^{2}Z\\ & & +H_{E}c_{11}\cos\dot{X}\cos X+H_{E}c_{12}\cos\dot{X}\cos Y+H_{E}c_{13}\cos\dot{X}\cos Z\\ & & +H_{E}c_{21}\cos\dot{Y}\cos X+H_{E}c_{22}\cos\dot{Y}\cos Y+H_{E}c_{23}\cos\dot{Y}\cos Z\\ & & +H_{E}c_{31}\cos\dot{Z}\cos X+H_{E}c_{32}\cos\dot{Z}\cos Y+H_{E}c_{33}\cos\dot{Z}\cos Z. \end{array}$$

This is the 18-term T–L equation.

As we have

(6) $$\cos^{2}Z=1-\cos^{2}X-\cos^{2}Y,$$

the $\cos^{2}Z$ term can be represented by the $\cos^{2}X$ and $\cos^{2}Y$ terms, and so it can be removed. Furthermore, considering the derivatives of the two sides of (6), namely

(7) $$\cos\dot{Z}\cos Z=-\cos\dot{X}\cos X-\cos\dot{Y}\cos Y,$$

the $\cos\dot{Z}\cos Z$ term can be removed too. Lastly, the 16-term T–L model is built.

### 2.2. Analysis of Multicollinearity

Section 2.1 indicates that the variables of the T–L model are formed by the direction cosines of the magnetic field of the earth. These cosines can be calculated from the aircraft attitude. Let θ and ϕ represent the magnetic heading and the dip angle, respectively, and λ, ψ, and Ω represent the pitch, roll, and yaw angles, respectively. Then, the direction cosines of the magnetic field of the earth can be represented as below [6]. For pitches:

(8) $$\begin{array}{c} \cos X=\cos\phi \sin\theta ,\\ \cos Y=\cos\phi \cos\theta \cos\lambda +\sin\phi \sin\lambda ,\\ \cos Z=\sin\phi \cos\lambda -\cos\phi \cos\theta \sin\lambda . \end{array}$$

For rolls:

(9) $$\begin{array}{c} \cos X=\cos\phi \sin\theta \cos\psi +\sin\phi \sin\psi ,\\ \cos Y=\cos\phi \cos\theta ,\\ \cos Z=\sin\phi \cos\psi -\cos\phi \sin\theta \sin\psi . \end{array}$$

For yaws:

(10) $$\begin{array}{c} \cos X=\cos\phi \sin\theta \cos\Omega -\cos\phi \cos\theta \sin\Omega ,\\ \cos Y=\cos\phi \cos\theta \cos\Omega +\cos\phi \sin\theta \sin\Omega ,\\ \cos Z=\sin\phi . \end{array}$$

As θ is a certain value in each heading, some simplified expressions can be deduced from Equations (8)–(10). In Table 1 and Table 2, we list the model variables and the relevant simplified expressions when the heading is north and east, respectively. The cases when the heading is south and west are very similar to Table 1 and Table 2, respectively.

As λ, ψ, and Ω are small angles, the following assumptions are established:

(11) cosλ=cosψ=cosΩ=1,

and

(12) $$\sin^{2}\lambda =\sin^{2}\psi =\sin^{2}\Omega =0.$$

According to Equations (11) and (12), we have some linear correlations in Table 1:

(13) $$\cos Z=-\frac{\cos\phi }{\sin\phi }\cos Y+\frac{1}{\sin\phi },$$

(14) $$\cos^{2}Y=2\cos\phi \cos Y-\cos^{2}\phi ,$$

(15) $$\cos Y\cos Z=\frac{\left(\sin^{2}\phi -\cos^{2}\phi \right)}{\sin\phi }\cos Y+\frac{\cos^{3}\phi }{\sin\phi },$$

(16) cosXcosY=cosϕcosX,and

(17) cosXcosZ=sinϕcosX.

These equations indicate that when the heading is the north, $A_{2}$, $A_{3}$, $A_{7}$, and $A_{8}$ correlate with each other, and $A_{1}$, $A_{5}$, and $A_{6}$ also correlate with each other. These correlations lead to multicollinearity. For the T–L model, the correlations mean that some coefficients can be represented by other coefficients. Hence, these variables are ineffective in calculating the magnetic interference field and negative for compensation.

When the heading is east, multicollinearity also exists; however, the correlations are caused by different sources. From Table 2, the linear correlations are as below:

(18) $$\cos Z=-\frac{\cos\phi }{\sin\phi }\cos X+\frac{1}{\sin\phi },$$

(19) $$\cos^{2}X=2\cos\phi \cos X-\cos^{2}\phi ,$$

(20) $$\cos X\cos Z=\frac{\sin^{2}\phi -\cos^{2}\phi }{\sin\phi }\cos X+\frac{\cos^{3}\phi }{\sin\phi },$$

(21) cosXcosY=cosϕcosY,and

(22) cosYcosZ=sinϕcosY,

where $A_{1}$, $A_{3}$, $A_{4}$, and $A_{6}$ correlate with each other, and $A_{2}$, $A_{5}$, and $A_{8}$ correlate with each other.

The comparison of these two cases indicates that the variables causing the multicollinearity are different. In other words, the variables causing multicollinearity are related to the flight heading.

### 2.3. The Multimodel Method

Based on the theory of regression, multicollinearity renders the variances of the least squares (LS) estimates large, and setting some coefficients to zero can improve the estimate accuracy [18]. Thus, reducing some of the correlated variables can be effective in mitigating the multicollinearity of the T–L model. However, in Section 2.2, we found that the correlated variables are not the same in different headings. So treating the different heading cases in different ways can suppress the influence of the multicollinearity more effectively. In this paper, we propose a method to select a subset of the variables to build a sub-model for a specific heading.

We choose the variance inflation factors (VIFs), which are indicators of multicollinearity [19] and defined as the diagonal elements of ${\left(A^{T}A\right)}^{-1}$, to be the criteria for determining the selected variables. For the purpose of retaining the variables that contribute less to multicollinearity, the selection manner is designed to exclude the variables that have larger VIFs because the larger the VIF is, the more the relevant variable contributes to the multicollinearity and should not be selected. If the variables that have larger VIFs are excluded, the multicollinearity of the shrunk model will be weakened.

**Figure 2:**
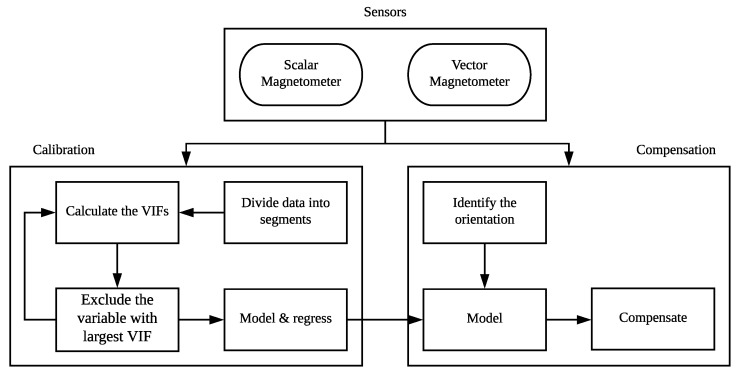
Procedures for calibration and compensation. VIF—variance inflation factor.

The implementation procedures of our method are shown in Figure 2 and described as follows. In the calibration stage, we divide the raw data collected during a calibration flight into four segments, according to the flight headings. For each segment, we select a variable set, by comparing the VIFs, to build the sub-model and estimate the coefficients. The estimation method is still the LS method. From the calibration flight, four sets of coefficients are obtained. In the compensation stage, first, the current heading needs to be identified to choose the relevant set of coefficients, and then the compensation is implemented.

Note that in the calibration flight, large maneuvers cause extra displacements of the magnetometer. Due to the gradient of the magnetic field of the earth, the measurement of the magnetic field will be also disturbed by these displacements, besides the aircraft’s magnetic field. To abandon the impact caused by the displacement of the magnetometer in the magnetic field of the earth, the gradient should be corrected. According to [7], we add a three-term gradient model after each shrunk T–L model. If the earth’s magnetic gradient is written as $\left(\begin{array}{ccc} g_{N} & g_{E} & g_{Z} \end{array}\right)$, where $g_{N}$, $g_{E}$, and $g_{Z}$ represent the gradient components of the magnetic north, magnetic east, and the local vertical, respectively, the variation in the magnetic field caused by the displacement of the magnetometer can be represented by

(23) $$H_{G}=g_{N}d_{N}+g_{E}d_{E}+g_{Z}d_{Z},$$

where $d_{N}$, $d_{E}$, and $d_{Z}$ represent the magnetometer displacements in the magnetic north, the magnetic east, and the local vertical, respectively. Finally, the sub-model corresponding to the specific flight heading is the combination of the shrunk T–L model and the gradient model.

## 3. Experiment

### 3.1. Implementation

**Figure 3:**
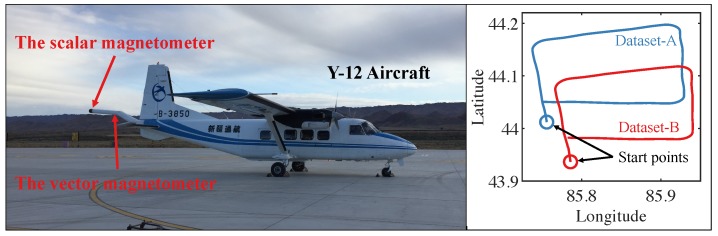
The aircraft used in the experiment.

The experiment was implemented in a Y-12 aircraft with the magnetometers installed in the tail boom. As shown in Figure 3, the scalar magnetometer and the three-axis vector magnetometer were equipped at the pole and the middle of the tail boom, respectively. The scalar magnetometer was a homemade helium magnetometer with sensitivity <0.001nT/√Hz@1Hz; its principle can be seen in [20,21].

As shown on the right of Figure 3, two datasets were adopted to test the compensation performance of the proposed method. They were labeled Dataset-A and Dataset-B. The flight headings were sorted by north, east, south, and west. The maneuvers were operated in the order of roll, pitch, and yaw. The flight altitude was about 3000 m. In each heading, the flight length was about 2 to 3 min.

Table 3 shows the process of selecting the variables in the north heading case, where FVS is short for full variable set and EVS is short for excluded variable set. In the beginning, we entered all of the 16 variables (FVS) and listed their VIFs. The largest VIF belonged to $A_{2}$, namely cosY. In other words, $A_{2}$ contributed the most to the multicollinearity and should be excluded. Subsequently, the other variables (EVS-1) were entered. This time, many of the VIFs were much smaller because the relevant variables exhibited strong collinearity with cosY. The largest VIF belonged to $A_{7}$ ($\cos^{2}Y$), which was also excluded. In EVS-2, the excluded variable was $A_{1}$ (cosX) and in EVS-3 it was $A_{13}$ ($\cos Y\cos\dot{Y}$). After this, all of the VIFs were much smaller.

The east case was similar to the north case. Table 4 shows the VIFs of the east segment in the same dataset. In the beginning, although the VIF of cosZ ($A_{3}$) was larger, we retained cosZ and excluded the second largest, cosX ($A_{1}$); this was because $\cos^{2}Z$ and $\cos Z\cos\dot{Z}$ are already excluded in the 16-term model, and so deleting cosZ causes the loss of too much information about the *Z*-axis. By deleting $A_{2}$ and $A_{4}$, the VIF of cosZ is small. Subsequently, cosY ($A_{2}$), $\cos^{2}X$ ($A_{4}$), and $\cos X\cos\dot{X}$ ($A_{9}$) were excluded, identically to the excluded variables in the north case but with the former *Y* replaced by *X*. The *X*-axis points north if the aircraft is oriented to the east, instead of the *Y* axis. These results indicate that the excluded variable set is stable.

In some data segments, the case where cosZ causes the largest VIF happens sometimes. However, to avoid losing too much information, we retain cosZ and exclude the variable that causes the second-largest VIF. Taking no account of this case, the results of Table 3 and Table 4 are stable. In the south and west segments, the subsets are the same as in the north and east segments, respectively. In other datasets, these results are reiterated. With these subsets, we build a specific model for each orientation.

**Figure 4:**
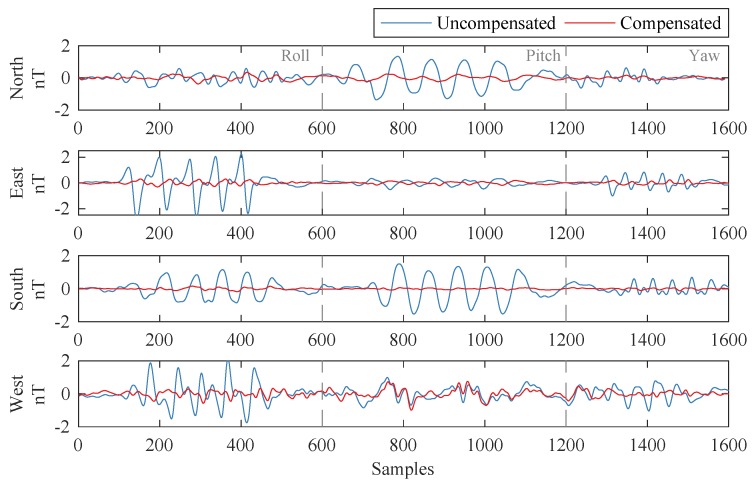
Compensation results.

Figure 4 shows the compensation results of our proposed method, where the selected subset was EVS-4; the coefficients calculated from Dataset-A were used to compensate Dataset-B. The FOMs, which are defined as the sums of peak-to-peak values in 12 maneuvers, were 26.6769 nT and 7.0414 nT before and after compensation, respectively. The standard deviations (STDs) of the uncompensated and compensated signals were 0.5230 nT and 0.1187 nT. The improvement ratio (IR), which is defined as the ratio between these two STDs, was 4.4054.

### 3.2. Comparison with Conventional Methods

**Figure 5:**
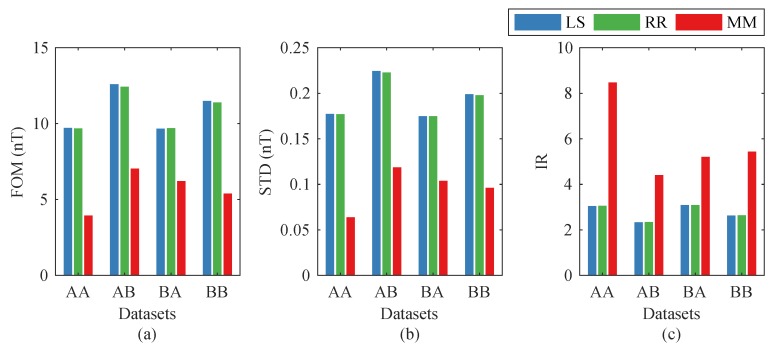
Comparisons between the three compensation methods: (**a**) figure-of-merit (FOM), (**b**) standard deviation (STD), and (**c**) improvement ratio (IR). LS—least squares; RR—ridge regression; MM—multimodel.

To verify the performance of the multimodel (MM) method proposed in this paper, we compare it with two conventional methods: the traditional LS method and the RR method. The three methods were respectively tested on the two datasets. Figure 5 shows the comparison results, where the FOMs and IRs of the three methods are exhibited. In this figure, the labels of the *x*-axis indicate the relationships between the two datasets. The first letter represents the calibration dataset, and the second one represents the compensation dataset (e.g., AB means using the coefficient set calculated from Dataset-A to compensate Dataset-B). From Figure 5, we find that in each test, the LS method and the RR method had the worst performances, and the MM method showed an obvious improvement.

**Figure 6:**
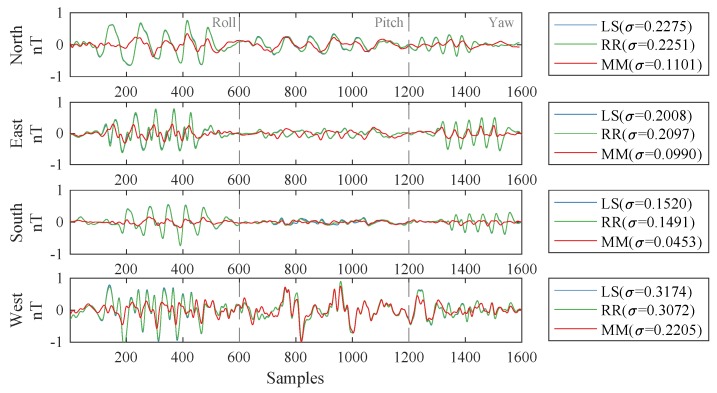
Compensation results of the three compensation methods. In the legends, σ represents the standard deviation (STD), with unit of nT.

Figure 6 shows the compensation results of the three methods in the four flight headings of Dataset-B, where the coefficient sets were calculated from Dataset-A. The figure indicates that the conventional LS and RR methods, based on the 16-term model, gave similar results, and the proposed MM method performed better in compensating for the magnetic interference field. The peak-to-peak values are showed in Table 5. In each box, the four numbers represent the peak-to-peak values of uncompensated, LS-compensated, RR-compensated, and MM-compensated signals, respectively. For these methods, The improvement factors of the FOMs were 2.1192, 2.1448, and 3.7886, respectively.

### 3.3. Compensations in Level Flights

**Figure 7:**
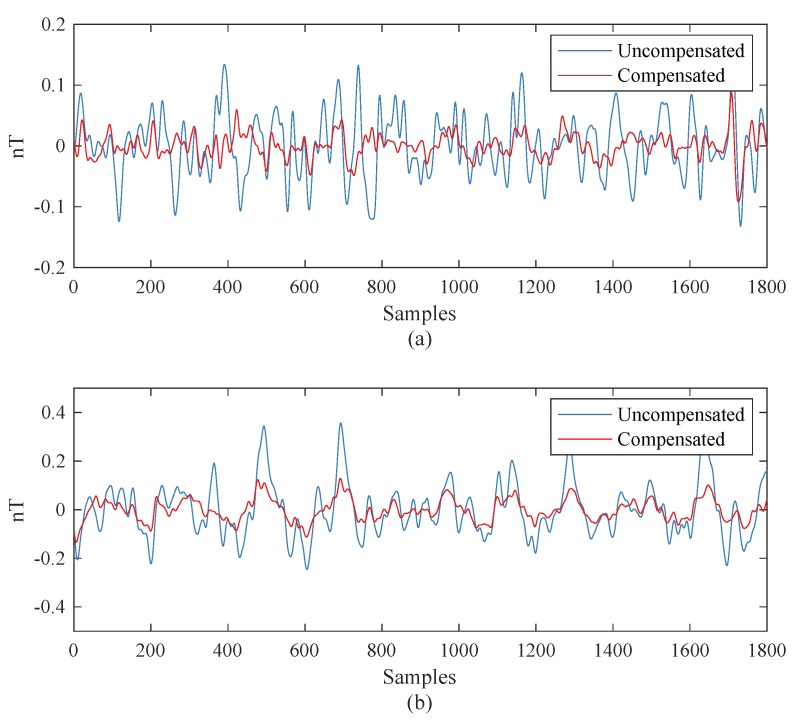
Compensation results: (**a**) Line-A and (**b**) Line-B.

The FOM flights mentioned above, containing different headings and different large maneuvers, are implemented for calibrating the coefficients and evaluating the compensation ability. Here, we present two tests on level flights, which are closer to the practical conditions. These flights, named Line-A and Line-B, were oriented to the North and South, respectively, with a length of 3 min. The coefficients applied here were those calculated from Dataset-A. Figure 7 shows the compensation results. The STD of Line-A reduced from 0.0506 nT to 0.0206 nT and that of Line-B reduced from 0.1076 nT to 0.0452 nT. The IRs were 2.4565 and 2.3835, respectively. This figure illustrates that the proposed MM method can help to measure the magnetic field more accurately with an airborne magnetometer.

We also compared the MM method with the two conventional methods on Line-A and Line-B. Table 6 shows the results of the comparisons between the three methods. From this, we find that on both datasets, the proposed MM method gave better results than the other two conventional methods.

### 3.4. Compensation in Non-Standard Headings

The experimental results above have proved that in the standard headings (north, east, south, and west), the MM method is effective in aeromagnetic compensation and better than the conventional methods based on the 16-term T–L model. However, in practice, sometimes the flight heading is not standard. Hence, it is necessary to test the performance of the proposed MM method in non-standard headings.

**Figure 8:**
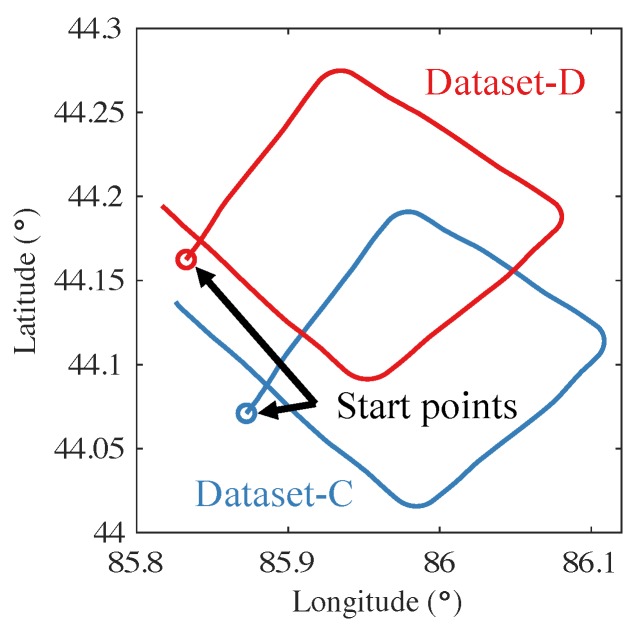
The tracks of Dataset-C and Dataset-D.

**Figure 9:**
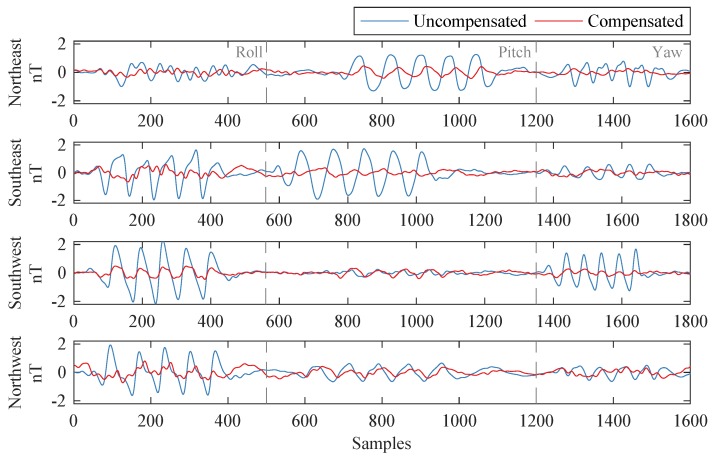
The compensated signal of Dataset-C. The coefficients are calculated from Dataset-C.

Figure 8 shows the tracks of other datasets, labeled as Dataset-C and Dataset-D. These two datasets were collected while the aircraft was flown towards the Northeast, Southeast, Southwest, and Northwest. The aircraft, the magnetometer, and the other sensors were unchanged. The compensation result is shown in Figure 9. The FOM was reduced from 28.3546 nT (uncompensated) to 4.4953 nT (compensated), the STD was reduced from 0.5813 nT (uncompensated) to 0.0902 nT (compensated), and the IR was 6.4470.

**Figure 10:**
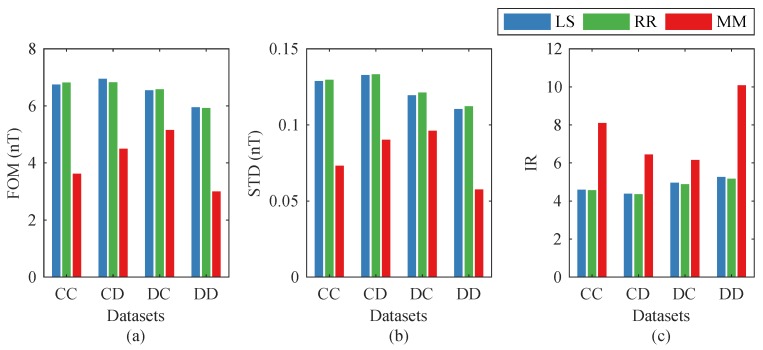
Comparisons between the three compensation methods in non-standard headings: (**a**) figure-of-merit (FOM), (**b**) standard deviation (STD), and (**c**) improvement ratio (IR).

We also compared our MM method with the LS and RR methods. The comparison results are shown in Figure 10. It indicates that the MM method is still better than the traditional LS and RR methods in the non-standard flight headings.

## 4. Conclusions

Magnetometers are usually equipped aboard an aircraft in many applications, such as geophysical exploration. As the magnetic field caused by the aircraft interferes with the measurements, an aeromagnetic compensation method should be applied. Most aeromagnetic compensation methods are based on the T–L model and are restricted by its multicollinearity. Herein, we have found that the model variables causing the multicollinearity differ according to the flight heading, based on which we proposed a multimodel method to mitigate the multicollinearity. This method built different sub-models for different headings by selecting the variables with smaller VIFs. In the real flight experiments, the MM method reduced the FOM from 26.6769 to 7.0414. The improvement factor is 3.7886, higher than the factors yielded by two conventional methods (the LS and RR methods), which were 2.1192 and 2.1448, respectively. In the level flight tests, the MM method reduced the STDs by about 2.4 times.

## Figures and Tables

**Table 1:** Model variables in the north heading.

Variable	Expression	Pitch	Roll	Yaw
$A_{1}$	cosX	0	sinϕsinψ	−cosϕsinΩ
$A_{2}$	cosY	cosϕcosλ+sinϕsinλ	cosϕ	cosϕcosΩ
$A_{3}$	cosZ	sinϕcosλ−cosϕsinλ	sinϕcosψ	sinϕ
$A_{4}$	$\cos^{2}X$	0	$\sin^{2}\phi \sin^{2}\psi$	$\cos^{2}\phi \sin^{2}\Omega$
$A_{5}$	cosXcosY	0	sinϕcosϕsinψ	$-\cos^{2}\phi \sin\Omega \cos\Omega$
$A_{6}$	cosXcosZ	0	$\sin^{2}\phi \sin\psi \cos\psi$	−sinϕcosϕsinΩ
$A_{7}$	$\cos^{2}Y$	$\cos^{2}\phi \cos^{2}\lambda +2\sin\phi \cos\phi \sin\lambda \cos\lambda +\sin^{2}\phi \sin^{2}\lambda$	$\cos^{2}\phi$	$\cos^{2}\phi \cos^{2}\Omega$
$A_{8}$	cosYcosZ	$\sin\phi \cos\phi \cos^{2}\lambda +\left(\sin^{2}\phi -\cos^{2}\phi \right)\sin\lambda \cos\lambda -\sin\phi \cos\phi \sin^{2}\lambda$	sinϕcosϕcosψ	sinϕcosϕcosΩ
$A_{9}$	$\cos X\cos\dot{X}$	0	$\sin^{2}\phi \sin\psi \cos\psi$	$\cos^{2}\phi \sin\Omega \cos\Omega$
$A_{10}$	$\cos X\cos\dot{Y}$	0	0	$\cos^{2}\phi \sin^{2}\Omega$
$A_{11}$	$\cos X\cos\dot{Z}$	0	$-\sin^{2}\phi \sin^{2}\psi$	0
$A_{12}$	$\cos Y\cos\dot{X}$	0	sinϕcosϕcosψ	$-\cos^{2}\phi \cos^{2}\Omega$
$A_{13}$	$\cos Y\cos\dot{Y}$	$\sin\phi \cos\phi \cos^{2}\lambda +\left(\sin^{2}\phi -\cos^{2}\phi \right)\sin\lambda \cos\lambda -\sin\phi \cos\phi \sin^{2}\lambda$	0	$-\cos^{2}\phi \sin\Omega \cos\Omega$
$A_{14}$	$\cos Y\cos\dot{Z}$	$-\cos^{2}\phi \cos^{2}\lambda -2\sin\phi \cos\phi \sin\lambda \cos\lambda -\sin^{2}\phi \sin^{2}\lambda$	−sinϕcosϕsinψ	0
$A_{15}$	$\cos Z\cos\dot{X}$	0	$\sin^{2}\phi \cos^{2}\psi$	−sinϕcosϕcosΩ
$A_{16}$	$\cos Z\cos\dot{Y}$	$\sin^{2}\phi \cos^{2}\lambda -2\sin\phi \cos\phi \sin\lambda \cos\lambda +\cos^{2}\phi \sin^{2}\lambda$	0	−sinϕcosϕsinΩ

**Table 2:** Model variables in the east heading.

Variable	Expression	Pitch	Roll	Yaw
$A_{1}$	cosX	cosϕ	cosϕcosψ+sinϕsinψ	cosϕcosΩ
$A_{2}$	cosY	sinϕsinλ	0	cosϕsinΩ
$A_{3}$	cosZ	sinϕcosλ	sinϕcosψ−cosϕsinψ	sinϕ
$A_{4}$	$\cos^{2}X$	$\cos^{2}\phi$	$\cos^{2}\phi \cos^{2}\psi +2\sin\phi \cos\phi \sin\psi \cos\psi +\sin^{2}\phi \sin^{2}\psi$	$\cos^{2}\phi \cos^{2}\Omega$
$A_{5}$	cosXcosY	sinϕcosϕsinλ	0	$\cos^{2}\phi \sin\Omega \cos\Omega$
$A_{6}$	cosXcosZ	sinϕcosϕcosλ	$\sin\phi \cos\phi \cos^{2}\psi +\left(\sin^{2}\phi -\cos^{2}\phi \right)\sin\psi \cos\psi -\sin\phi \cos\phi \sin^{2}\psi$	sinϕcosϕcosΩ
$A_{7}$	$\cos^{2}Y$	$\sin^{2}\phi \sin^{2}\lambda$	0	$\cos^{2}\phi \sin^{2}\Omega$
$A_{8}$	cosYcosZ	$\sin^{2}\phi \sin\lambda \cos\lambda$	0	sinϕcosϕsinΩ
$A_{9}$	$\cos X\cos\dot{X}$	0	$\sin\phi \cos\phi \cos^{2}\psi +\left(\sin^{2}\phi -\cos^{2}\phi \right)\sin\psi \cos\psi -\sin\phi \cos\phi \sin^{2}\psi$	$-\cos^{2}\phi \sin\Omega \cos\Omega$
$A_{10}$	$\cos X\cos\dot{Y}$	sinϕcosϕcosλ	0	$\cos^{2}\phi \cos^{2}\Omega$
$A_{11}$	$\cos X\cos\dot{Z}$	−sinϕcosϕsinλ	$-\cos^{2}\phi \cos^{2}\psi -2\sin\phi \cos\phi \sin\psi \cos\psi -\sin^{2}\phi \sin^{2}\psi$	0
$A_{12}$	$\cos Y\cos\dot{X}$	0	0	$-\cos^{2}\phi \sin^{2}\Omega$
$A_{13}$	$\cos Y\cos\dot{Y}$	$\sin^{2}\phi \sin\lambda \cos\lambda$	0	$\cos^{2}\phi \sin\Omega \cos\Omega$
$A_{14}$	$\cos Y\cos\dot{Z}$	$-\sin^{2}\phi \sin^{2}\lambda$	0	0
$A_{15}$	$\cos Z\cos\dot{X}$	0	$\sin^{2}\phi \cos^{2}\psi -2\sin\phi \cos\phi \sin\psi \cos\psi +\cos^{2}\phi \sin^{2}\psi$	−sinϕcosϕsinΩ
$A_{16}$	$\cos Z\cos\dot{Y}$	$\sin^{2}\phi \cos^{2}\lambda$	0	sinϕcosϕcosΩ

**Table 3:** Variance inflation factors (VIFs) in the north heading. FVS—full variable set; EVS—excluded variable set.

Variables			VIFs		
	FVS	EVS-1	EVS-2	EVS-3	EVS-4
$A_{1}$	9730.40	6640.77	**5119.77**	—	—
$A_{2}$	**159,672.01**	—	—	—	—
$A_{3}$	112,862.35	39,651.18	23.55	23.54	23.32
$A_{4}$	16,679.02	9875.18	21.07	20.81	20.78
$A_{5}$	1907.04	1312.74	1311.42	512.57	511.42
$A_{6}$	4727.15	3772.57	2369.45	509.71	507.73
$A_{7}$	36,281.72	**35,521.73**	—	—	—
$A_{8}$	26,334.11	526.00	20.67	20.67	20.49
$A_{9}$	626.94	626.33	623.44	619.19	15.07
$A_{10}$	1.60	1.60	1.60	1.46	1.43
$A_{11}$	2.51	2.49	2.47	2.46	2.35
$A_{12}$	488.07	488.07	487.06	482.17	443.24
$A_{13}$	1946.64	1945.84	1932.61	**1913.33**	—
$A_{14}$	1169.43	1168.18	1162.95	1154.38	25.10
$A_{15}$	483.08	483.08	481.86	477.28	436.85
$A_{16}$	510.18	510.11	504.93	499.07	10.90

**Table 4:** Variance inflation factors (VIFs) in the east heading.

Variables			VIFs		
	FVS	EVS-1	EVS-2	EVS-3	EVS-4
$A_{1}$	**397,320.73**	—	—	—	—
$A_{2}$	27,811.26	**27,266.38**	–	—	—
$A_{3}$	(400,873.05)	2809.42	2763.78	3.38	3.38
$A_{4}$	35,405.72	3384.67	**3336.12**	—	—
$A_{5}$	3626.27	3534.16	37.41	37.39	37.29
$A_{6}$	51,546.73	49.68	49.51	3.37	3.36
$A_{7}$	138.65	3.30	3.03	2.61	1.79
$A_{8}$	11,488.87	11,299.04	39.48	39.41	39.09
$A_{9}$	328.74	324.86	222.25	**219.56**	—
$A_{10}$	71.27	67.20	66.60	66.60	66.16
$A_{11}$	104.81	104.04	75.54	73.93	4.41
$A_{12}$	22.37	22.35	19.98	18.54	18.47
$A_{13}$	1.60	1.60	1.49	1.45	1.39
$A_{14}$	19.68	19.67	16.97	15.74	15.72
$A_{15}$	98.89	97.12	61.96	61.87	5.20
$A_{16}$	69.11	65.23	64.58	64.36	63.81

**Table 5:** The peak-to-peak values of uncompensated (UN), LS-compensated, RR-compensated, and MM-compensated signals (UN|LS|RR|MM).

Maneuver	North	East	South	West	Sum
Roll	1.19|1.39|1.40|0.72	5.11|1.33|1.41|0.61	2.01|1.29|1.26|0.34	3.88|2.00|1.89|0.86	12.19|6.00|5.97|2.53
Pitch	2.71|0.66|0.62|0.47	0.89|0.34|0.32|0.36	3.04|0.24|0.23|0.15	1.83|1.86|1.85|1.74	8.47|3.09|3.02|2.73
Yaw	1.25|0.67|0.65|0.31	1.84|1.03|1.06|0.42	1.10|0.69|0.67|0.17	1.82|1.11|1.07|0.88	6.01|3.49|3.45|1.78
Sum	5.15|2.72|2.67|1.50	7.84|2.70|2.80|1.39	6.15|2.21|2.16|0.66	7.54|4.96|4.81|3.79	26.68|12.59|12.44|7.04

**Table 6:** The STDs and IRs of the three methods on Line-A and Line-B.

Dataset		STD (nT)			IR	
	LS	RR	MM	LS	RR	MM
Line-A	0.0223	0.0216	0.0206	2.2695	2.3452	2.4565
Line-B	0.0579	0.0594	0.0452	1.8594	1.8124	2.3835
